# Improvement of Growth Rate in *In Vitro* Culture of *Paphiopedilum primulinum* M. W. Wood & P. Taylor and *Paphiopedilum glaucophyllum* J. J. Smith using Banana Enrichment Media

**DOI:** 10.21315/tlsr2024.35.3.5

**Published:** 2024-10-07

**Authors:** Safitri, Dyah Carinae Yalapuspita, Elizabeth Handini, Popi Aprilianti, Yupi Isnaini, Endang Semiarti

**Affiliations:** 1 Graduate School, Universitas Gadjah Mada, Jl. Teknika Utara, Sleman 55281, Yogyakarta, Indonesia; 2 Research Center for Plant Conservation and Botanic Gardens, National Research and Innovation Agency, Techno Park of Dr. (H.C.) Ir. Soekarno, Jl. Raya Jakarta – Bogor KM 46, Cibinong, Bogor 16911, West Java, Indonesia; 3 Faculty of Biology, Universitas Gadjah Mada, Jl. Teknika Selatan, Sekip Utara, Sleman 55281, Yogyakarta, Indonesia

**Keywords:** *In vitro* Culture, *Paphiopedilum primulinum*, *Paphiopedilum glaucophyllum*, Treatment Media

## Abstract

*Paphiopedilum primulinum* and *Paphiopedilum glaucophyllum* have unique labellum colour and shaped like lady’s slippers. These orchids are from the Cochlopetalum section, which is exclusively found in Sumatra and Java. There are so many people that desire to collect these plants illegally. Due to extensive commercial exploitation, *Paphiopedilum* is in danger of going extinct. Tissue culture techniques are utilised to conserve threatened orchid germplasm in a short time. The success of the *in vitro* culture depends on the accuracy of the basic media composition used. The Ambon Lumut banana (ALB) can accelerate plant growth and cell division. Banana added to the culture medium was prepared by mashing the ripe flesh (3.5 months old) using a mortar. This research aims to investigate the effect of banana homogenate supplemented media for the orchids *P. primulinum* and *P. glaucophyllum* based on the parameters of difference of plant height (calculated from the base of the stem to the tip of the plant stem), number of leaves, and number of roots. The measurement method was carried out using a ruler with a centimetre scale. Observations and documentation were carried out once a week for 7 weeks after planting (WAP) for *P. primulinum* and *P. glaucophyllum*. The results showed that ½ Murashige and Skoog (MS) + ALB homogenate is a better medium for *P. primulinum* and *P. glaucophyllum* growth than media without banana homogenate. The highest values of plant height, leaf growth and root growth of *P. primulinum* with banana homogenate were 0.44 cm, 0.63 leaves, and 0.50 roots, respectively. The highest values of plant height and leaf growth of *P. glaucophyllum* were 0.75 cm and 1.90 leaves, respectively. Culture medium added banana homogenate was able to support the propagation of plants, some of which are returned to nature and others used for industrial purposes (conventionally cultivated by the community).

**Highlights**
- The impact of supplementing the medium with banana homogenate on the orchid species *Paphiopedilum primulinum* and *Paphiopedilum glaucophyllum* was determined by comparing the plants’ heights (measured from the base to the tip of the stem), leaf count and root count.
- ½ Murashige and Skoog (MS) + *Ambon Lumut* banana (ALB) homogenate is a better medium for *P. primulinum* and *P. glaucophyllum* growth than media without banana homogenate.
- Culture medium added banana homogenate was able to support the propagation of plants.

## INTRODUCTION

**Figure 1:**
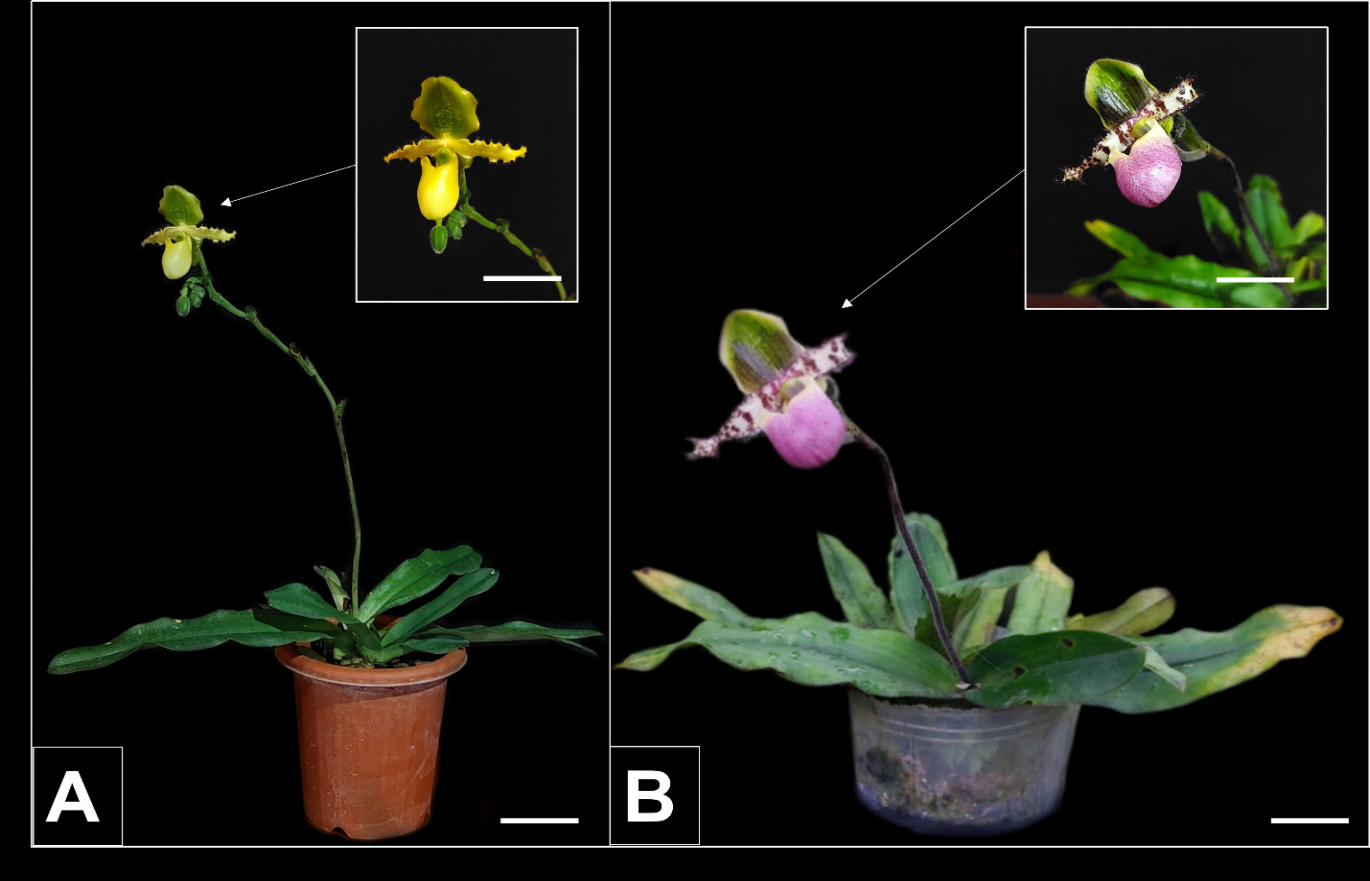
Habitus of (A) *P. primulinum* and (B) *P. glaucophyllum*. *Note*: Bars = 5 cm.

Orchids are ornamental plants that have high economic value because their morphology and colour are diverse and attractive (Luo *et al*. 2021). One of the orchid genera that has a unique pouch-shaped lip of a lady’s slipper-like labellum is the genus *Paphiopedilum*, which is included in the Appendix 1 category based on the Convention of International Trades in Endangered Species of Wild Fauna and Flora (CITES). The genus *Paphiopedilum* is the most widely cultivated and hybridised genus of orchids. Overexploitation or illegal activities, natural disasters, conversion of land into settlements and habitat destruction are the causes of *Paphiopedilum* population decline in nature (Kaur & Bhutani 2013; Sindiya *et al*. 2018). According to Azmi and Wiendi (2015), it takes a long time to produce *Paphiopedilum* seeds in large quantities. This plant reproduces by producing saplings, and it takes two to three years to produce saplings (Birk 1983). Two of the endangered *Paphiopedilum* species are *Paphiopedilum primulinum* and *P. glaucophyllum*. *P. primulinum* is a pouch-shaped lip of a lady’s slipper orchid endemic to Sumatra (southern Aceh), and the abundance of this orchid species has decreased significantly in the last few decades (Fig. 1A). In addition, another *Paphiopedilum* species that is threatened with extinction is *P. glaucophyllum* J. J. Smith, which is a plant native to West Java that was discovered and introduced by J. J. Smith in 1900 (Fig. 1B) (Azmi & Wiendi 2015; Destri & Ismaini 2018).

Tissue culture techniques are used to conserve rare and endangered orchid germplasm (Pant *et al*. 2022). The success of *in vitro* culture is influenced by the growth media and growth effect substances (Plant growth regulators [PGRs]) used (Chaudhary & Prakash 2019). Tissue culture media generally contain macro- and micronutrients, vitamins, amino acids, sucrose and growth regulators. A medium pH that is too low (<4.5) or too high (>7.0) can inhibit or stop the growth and development of *in vitro* cultures (Widiastoety *et al*. 2005). There are several common types of basic *in vitro* culture media, namely, MS – Murashige and Skoog (used on almost all types of plants), VW – Vacin and Went, NP – New Phalaenopsis and KC – Knudson C. Organic substances can enhance the process of development and regeneration of *in vitro* plants (Utami & Hariyanto 2020).

*Ambon Lumut* banana (ALB) was used because it has higher Ca (7.22 mg), and vitamin C (19.10 mg) content than *Ambon* and *Raja* bananas. Bananas are often added to *in vitro* culture media, and the addition of 25 g/L ALB into the culture medium stimulated the multiplication of *Phalaenopsis fuscata* shoots and leaves (Garvita & Handini 2011). Nurfadilah *et al*. (2018) reported that ALB extract had the highest glucose content compared to other types of bananas. Glucose can be used as an energy source to stimulate cell division and promote cell differentiation, as well as trigger shoot growth. There are no studies yet of plantlet growth of *P. primulinum* and *P. glaucophyllum* orchids in relation to the addition of banana extract into culture media. Therefore, it is necessary to research the growth of *P. primulinum* and *P. glaucophyllum* orchids cultured in various types and concentrations of media (½ MS, ½ MS + ALB, MS, ½ NP, NP, KC and VW) *in vitro*.

## MATERIALS AND METHODS

### Plant Materials, Growth Conditions and Culture Conditions

The plant materials used in this research were 1-year-old *P. primulinum* and 2-year-old *P. glaucophyllum* orchid plantlets from the Research Center for Plant Conservation and Botanical Gardens, Indonesian Institute of Sciences BRIN (in-kind support used in this research). Plantlets were cultured on ½ MS, ½ MS + ALB, MS, ½ NP, NP, KC and VW treatment media (Merck Darmstadt, Germany) without the addition of growth regulators (PGR). The bananas used are ripe banana flesh that are about 3.5 months old. Banana homogenate was prepared by weighing the peeled banana at 20 g/L. The banana was mashed using a blender or mortar, added to ½ MS media and homogenised. Plantlets were planted in culture bottles containing treatment media using sterile tweezers and then placed in petri dishes to be cleaned of residual media. Cultures were maintained in treatment of light for 24 h and at a temperature of 25°C with 70% humidity.

#### Nutritional assessment of ALB

The nutritional content of ALB was assessed using the Atomic Absorption Spectroscopy (AAS) test method at the Laboratory of Food and Agricultural Product Technology, Faculty of Agricultural Technology, Universitas Gadjah Mada and the Integrated Research and Testing Laboratory, Universitas Gadjah Mada.

### Measurement of Height and Leaf and Root Growth of *P. primulinum* and *P.glaucophyllum*

The parameters observed in this study were plant height (measured from the base of the stem to the tip of the main stem), the number of leaves, and the number of roots growing on each plantlet. The tool used to measure plantlet height is a ruler with a centimetre scale. Growth was observed and documented once a week. Observations were made for 7 weeks after planting (WAP) for *P. primulinum* and *P.glaucophyllum*.

### Research Design

Due to the limited number of plants available as samples—considering that the plants included were uncommon species classified as endangered—only three repetitions of the experiment were conducted. In the experiments, three bottles were used, and each bottle contained one plantlet (each plantlet was referred to as one replicate). The design used in this study was a completely randomised design that was analysed by statistical ANOVA test through SPSS IBM Statistics 25. If there were differences, the test was continued with Duncan’s multiple distance test 5%.

## RESULTS AND DISCUSSION

### The Growth Response of *P. primulinum* and *P. glaucophyllum* Plantlets

The results showed that *P. primulinum* and *P. glaucophyllum* plantlets cultured on various types and concentrations of treatment media showed different growth parameters for plant height, leaf growth, and root growth. Based on ANOVA, which was further analysed using Duncan’s 5% test, the increases in plant height, leaf growth, and root growth were significantly different. This is shown in Table 1.

### Nutrient Value of ALB

Bananas are often used as an organic additive in *in vitro* culture media. They are rich in various natural growth regulators, vitamins, and minerals. One type of banana that is supplemented into media is ALB. The nutritional values of ALB are shown in Table 2.

### Plantlet Development of *P. primulinum* and *P. glaucophyllum*

**Figure 2:**
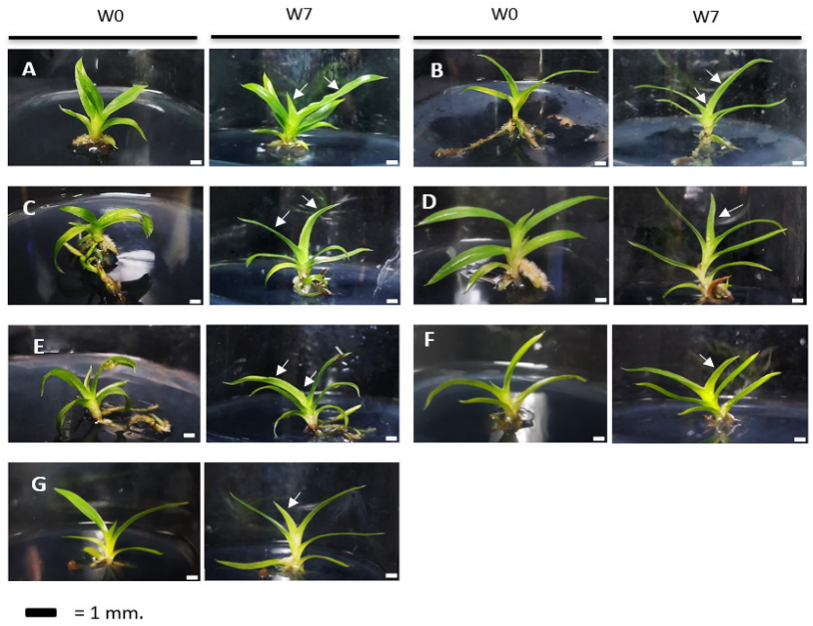
Plantlet development of *P. primulinum* on various culture media at week 0 and week 7 for (A) ½ MS, (B) ½ MS + ALB, (C) MS, (D) ½ NP, (E) NP, (F) KC, and (G) VW, respectively. *Notes*: Bars = 1 cm. The white arrows represent the growth of new leaves.

**Figure 3:**
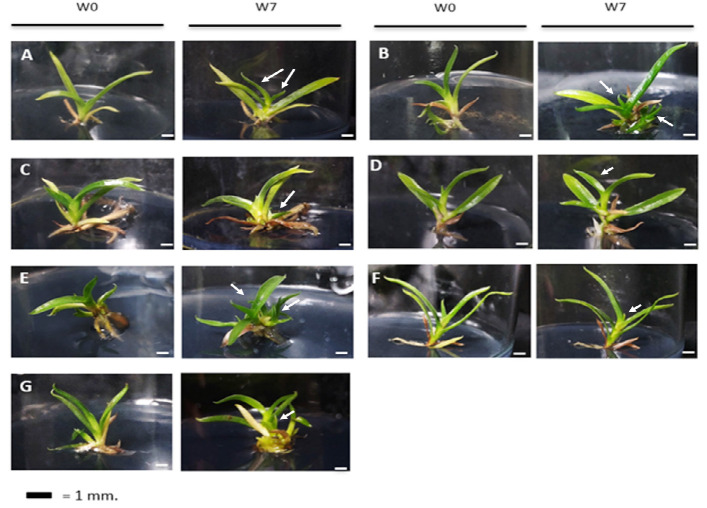
Plantlet development of *P. glaucophyllum* on various culture media at week 0 and week 7 for (A) ½ MS, (B) ½ MS + ALB, (C) MS, (D) ½ NP, (E) NP, (F) KC, and (G) VW, respectively. *Notes*: Bars = 1 cm. The white arrows represent the growth of new leaves.

The growth of new leaves is one response that is easily noticeable during the plantlets’ development. The growth of leaves on each plantlet, indicated by a white arrow, as seen in Figs. 2 and 3. In every culture medium, both species responded in the same way with the development of new leaves.

### The Growth Response of Plantlets

The type of media has a significant impact on the growth and development of plantlets (Tuhuteru *et al*. 2012). MS, NP, KC and VW are some types of basic media that are often used in orchid propagation and are available in full-strength and half-strength concentrations. The different contents of macro- and micronutrients in each type of medium have different effects on plant growth (Inkiriwang *et al*. 2016). Based on this study, half-strength MS (½ MS) media is the best treatment media; it is a complex media containing macronutrients, micronutrients and vitamins. When the concentration of the media changes, so do the nutrient components. As a result, different media concentrations or strengths can affect plant growth and organogenesis *in vitro* (Rezali *et al*. 2017). MS medium has been used for *in vitro* propagation of several orchid plants, including *Paphiopedilum insigne*, *Vanda pumila* Hook. f., Tolumnia Louise Elmore ‘Elsa’ (Deb & Jakha 2020). NP media was used to culture *Dendrobium lineale*, *Dendrobium phalaenopsis* and *Phalaenopsis amabilis* (Mustika & Semiarti 2021; Mose *et al*. 2020; Zulwanis *et al*. 2020).

The plant height of *P. glaucophyllum* was higher than that of *P. primulinum*, which is due to the different endogenous hormones contained in each species. The results of this study showed the highest leaf growth of *P. primulinum* in ½ MS media (0.67) and the highest leaf growth of *P. glaucophyllum* in ½ MS + ALB (1.90). The number of leaves formed on each planted explant is controlled by the balance and interaction between growth regulators both contained in the explant itself (endogenous) and absorbed from the media (exogenous).

Bhojwani and Razdan (1996) reported that ½ MS or ¼ MS medium produced better results in *Dendrocalamus* shoot propagation than full-strength MS medium. Furthermore, ½ MS medium is effective for propagating sugarcane orchids (*Grammatophyllum speciosum*), initiating shoot and root formation in *Phalaenopsis* protocorm-like bodies (PLB), and supporting shoot and root proliferation in the rare orchid *V. pumila* (Kriswanto *et al*. 2017; Maharjan *et al*. 2019; Sulichantini *et al*. 2021). In the previous study by Kaur and Bhutani (2012), *in vitro* propagation of *Cymbidium pendulum* supplemented with 50 g/L banana homogenate showed the highest number of induced shoots. Banana extract has potential as a sucrose replacement, and the sugar concentration in banana extract is high (27%). The addition of banana extract into the culture medium was able to produce the highest values for shoot induction and shoot height (Phillips *et al*. 2021). The banana homogenate contains a high concentration of potassium (K), which is essential to support the shoot regeneration of cultured plantlets (Lui *et al*. 2022).

Aside from having a positive effect on plant height, half-strength MS supplemented banana homogenate (½ MS + ALB) had the highest value of root growth on *P. primulinum*, with a value of 0.50 (Table 1). The highest value of root growth in *P. glaucophyllum* plantlets was shown in ½ MS and NP media. NP is a medium designed specifically for orchid germination that contains high nitrogen content that promote root initiation (Islam *et al*. 1998; Krisdianto *et al*. 2020). Because of the high nitrogen content in NP media, the media has a very positive effect on the root growth of *P. glaucophyllum*. Zeng *et al*. (2013) reported that ½ MS medium was more efficient for rooting *Withania coagulans* because its osmotic strength was lower than full-strength MS. Kartikaningrum *et al*. (2017) reported that MS media with complete nutrients causes browning in some orchid species such as *Rhyncostilis retusa*, *Dendrobium anosmum* and *Renanthera matutina* when cultured in the media.

Bananas contain a high concentration of carbohydrates and K, which are used as a source of sugar and macronutrients. *Ambon* banana and ALB are two types of bananas that are frequently used as supplements to *in vitro* growth media (Garvita & Handini 2011; Sulichantini *et al*. 2021). The glucose content of *Ambon* banana can stimulate shoot growth and cell division processes (Nurfadilah *et al*. 2018). Elements such as K, iron (Fe), and phosphorus (P) are also important for growth, so the ½ MS media treatment with banana pulp shows significant results. Table 2 shows the ALB content. ALB provided 298.8 mg of K per 100 g. Ranjha *et al*. (2022) reported that banana is an important source of K. A normal-sized banana can provide 350 mg of K. Each 100 g of *Musa acuminate* Colla provides 358 mg of K. In addition, 100 g of *Musa acuminate* Colla provides 5 mg of calcium (Ca) and 27 mg of magnesium (Mg). Ca is related to certain processes, such as membrane structure and function, ion uptake, reactions with growth regulators and enzymatic activation. The structural function of Ca is characterised by its use in the synthesis of new cell walls, especially the middle lamella, which separates newly dividing cells (Malavolta *et al*. 1997; Taiz & Zeiger 2006). Ca plays an important role in the transmission of hormonal signals; in media supplemented with Ca, the process of mitosis-cytokinesis increases. Ca is also able to stabilise cell membranes, plays a role in cell elongation and cell division, and affects cell pH.

Kaewubon and Meesawat (2014) showed that MS media supplemented with 50 g/L banana pulp can stimulate the development of *Paphiopedilum niveum* PLB into organised plant structures. *Ambon* banana contains hormones in the form of auxin and cytokinin, which can stimulate cell division and differentiation, which is beneficial for cell multiplication (Nurfadilah *et al*. 2018). Half-strength MS media supplemented banana pulp can increase the vitamin content of the media, which supports better growth. Thiamine, pyridoxine and nicotinic acid are three vitamins that are commonly used in plant *in vitro* culture (Hapsoro *et al*. 2018; Hasanah *et al*. 2014). Thiamine (vitamin B1) acts as a coenzyme in many metabolic pathways, particularly those involved in energy production and central metabolism, such as carbon assimilation and respiration (Fitzpatrick & Chapman 2020). According to Garvita and Handini (2011), banana extract contains thiamine, which plays a role in accelerating cell division of the root meristem. *Ambon* bananas, which are equivalent to Cavendish bananas, have thiamine levels of 0.04 mg/100 g (Garvita & Handini 2011; Sallolo *et al*. 2012). Thiamine at high concentrations promotes plant proliferation by inhibiting oxidative stress reactions that can damage plant tissue. This is the reason ½ MS + ALB media resulted in the greatest increase in plant height and root growth when compared to ½ MS media.

## CONCLUSION

Half-strength medium supplemented ALB (½ MS + ALB) was the best medium for the growth of *P. primulinum* orchids based on the parameters of plant height and root growth and the best medium for *P. glaucophyllum* orchids based on the parameters of plant height and leaf growth.

## Figures and Tables

**Table 1:** Average results of observations of plant height, leaf growth, and root growth on *P.primulinum* and *P. glaucophyllum* on various types and concentrations of treatmentmedia.

Media	*P. primulinum*			*P. glaucophyllum*		

	Difference of plantlet height (cm)	Number of leaves	Number of roots	Difference of plantlet height (cm)	Number of leaves	Number of roots
½ MS	0.41 ± 0.06^ab^	0.67 ± 0.13^a*^	0.42 ± 0.10^ab^	0.45 ± 0.08^bc^	0.64 ± 0.10^bc^	0.42 ± 0.19^a*^
½ MS+ALB*	0.44 ± 0.07^a*^	0.63 ± 0.13^a*^	0.50 ± 0.17^a*^	0.75 ± 0.08^a*^	1.9 ± 0.20^a*^	0.25 ± 0.09^ab^
MS	0.40 ± 0.09^abc^	0.67 ± 0.14^a^	0.17 ± 0.08^bc^	0.59 ± 0.10^ab^	0.58 ± 0.20^bc^	0.21 ± 0.08^ab^
½ NP	0.26 ± 0.04^bc^	0.33 ± 0.10^a^	0.13 ± 0.07^c^	0.27 ± 0.08^cd^	0.60 ± 0.10^bc^	0.12 ± 0.07^ab^
NP	0.24 ± 0.04^c^	0.50 ± 0.10^a^	0.00 ± 0.00^c^	0.32 ± 0.07^cd^	1.21 ± 0.40^b^	0.46 ± 0.17^a*^
KC	0.26 ± 0.03^bc^	0.46 ± 0.10^a^	0.38 ± 0.10^ab^	0.34 ± 0.06^cd^	1.26 ± 0.30^ab^	0.22 ± 0.08^ab^
VW	0.30 ± 0.04^abc^	0.33 ± 0.10^a^	0.13 ± 0.07^bc^	0.25 ± 0.04^cd^	0.25 ± 0.10^c^	0 ± 0.00^b^

*Notes*: ½ MS = half-strength MS media; ½ MS + ALB = half-strength MS media with banana homogenate; MS = full-strength MS media; ½ NP = half-strength NP media; NP = full-strength NP media; KC = full-strength KC media; VW = full-strength VW media. The symbol * represent the highest values from each parameter. Data in the same column followed by the same letters are not significantly different by Duncan’s test at *p* ≤ 0.05.

**Table 2:** Nutritional values of ALB in 100 g edible portion.

Nutritional content	ALB^a^	*Ambon* banana^b^	*Raja* banana^c^
Water	74.35%	75.70%	66.49%
Protein	0.92%	1.10%	1.51%
Fat	0.19%	0.20%	0.05%
Carbohydrate	23.32%	22.20%	31.13%
Ash	1.28%	0.80%	0.82%
Ca	7.22 mg	7.00 mg	–
Mg	16.25 mg	36.0 mg	–
K	298.9 mg	460.0 mg	350.0 mg
Vitamin C	19.10 mg	10.00 mg	16.45 mg

*Notes*:

a = The content analysis of ALB was conducted by the Test Laboratory of Food and Agricultural Product Technology, Faculty of Agricultural Technology, Universitas Gadjah Mada and the Integrated Research and Testing Laboratory, Universitas Gadjah Mada;

b = The nutritional content analysis of *Ambon* banana is based on Garvita and Handini (2011);

c = The nutritional content analysis of *Raja* banana is based on Hapsari and Lestari (2016).
